# Relation between bandgap and resistance drift in amorphous phase change materials

**DOI:** 10.1038/srep17362

**Published:** 2015-12-01

**Authors:** Martin Rütten, Matthias Kaes, Andreas Albert, Matthias Wuttig, Martin Salinga

**Affiliations:** 1 Institute of Physics 1A, RWTH Aachen University, Sommerfeldstrasse 14, 52074 Aachen, Germany; 2 IBM Research–Zurich, Säumerstrasse 4, 8803 Rüschlikon, Switzerland

## Abstract

Memory based on phase change materials is currently the most promising candidate for bridging the gap in access time between memory and storage in traditional memory hierarchy. However, multilevel storage is still hindered by the so-called resistance drift commonly related to structural relaxation of the amorphous phase. Here, we present the temporal evolution of infrared spectra measured on amorphous thin films of the three phase change materials Ag_4_In_3_Sb_67_Te_26_, GeTe and the most popular Ge_2_Sb_2_Te_5_. A widening of the bandgap upon annealing accompanied by a decrease of the optical dielectric constant ε_∞_ is observed for all three materials. Quantitative comparison with experimental data for the apparent activation energy of conduction reveals that the temporal evolution of bandgap and activation energy can be decoupled. The case of Ag_4_In_3_Sb_67_Te_26_, where the increase of activation energy is significantly smaller than the bandgap widening, demonstrates the possibility to identify new phase change materials with reduced resistance drift.

Looking at the commonly used memory hierarchy in computers, one finds a gap of more than three orders of magnitude between the access time of the memory level and the storage level^1^. The bottleneck between the volatile, fast, expensive DRAM and the non-volatile, slow, cheap FLASH storage is supposed to be bypassed by a storage class memory (SCM)^2^. Phase change memory (PCM) is currently the most promising SCM candidate, offering excellent scalability, endurance, read/write speed and non-volatility^3^,^4^. Such a PCM utilizes the ability of so-called phase change materials to switch between an amorphous and crystalline phase on a nanosecond timescale^5^. These two phases show a large contrast in electrical resistivity, which is employed to store the two states of a bit. To achieve a competitive storage density and thus lower the cost-per-bit, multiple levels of resistance have to be stored in one single cell by varying the ratio of amorphous and crystalline material. However, this multilevel storage concept is hindered by the so-called resistance drift, which appears as a slow but steady increase of resistivity over time in the amorphous phase. This phenomenon eventually leads to overlaps of closely spaced resistance levels and thus to decoding errors. Over the past years, several solutions for building a drift-resilient multilevel cell (MLC) have been presented^6^,^7^. These approaches accept the existence of resistance drift and correct for it by employing a more complex readout circuitry with the disadvantage of increasing latency. Understanding the underlying mechanisms of drift and finding a material composition showing comparatively low or even no drift would therefore be of great help to further increase the performance of PCM. In addition to the application as storage class memory, phase change materials recently attracted interest in the field of brain inspired computing, which aims at breaking the Von Neumann bottleneck in conventional computing^8^. Being more mature than technologies like resistive switching memory (RRAM) or conductive bridge memory (CBRAM), phase change memory was already implemented as a synaptic element in a large-scale neural network^9^,^10^. Improving the knowledge about resistance drift and MLC capability of PCM will be useful for this area of applications as well, because the gradual programming of resistance levels is a crucial requirement for synaptic devices^11^.

Like in any other disordered semiconductor, the amorphous phase of phase change materials lacks atomic long-range order. Therefore, the common band structure picture of a crystalline solid including a valence and conduction band must be complemented by localized states at the band edges and potentially additional defect states inside the bandgap. Within this framework, several attempts to explain the resistance drift can be found in literature, including e.g. the annealing of defect states^12^,^13^, the relaxation of mechanical stress^14^,^15^ or the formation of valence alternation pairs^16^. All of these processes lead to an increase of the bandgap during relaxation, which is supported by density functional theory calculations for several GeSbTe compositions^17^. With the Fermi level pinned close to the middle of the bandgap^18^, bandgap widening increases the energy difference between valence band edge and Fermi level, which is the activation energy for electrical conduction in these p-type semiconductors. Accordingly, an optically measured widening of the bandgap for GeTe during relaxation was suggested to be the reason for the increase in activation energy^19^. Supporting this assumption of a causal link between activation energy and bandgap widening, Fantini *et al.* even reported an equally strong absolute increase of both quantities upon relaxation for Ge_2_Sb_2_Te_5,_ one of the most popular phase change materials^20^. Eventually, Boniardi *et al.* completed the picture for this specific material by concluding that resistance drift can be fully traced back to the increase in the activation energy^21^. Until today, resistance drift is argued to be tightly correlated with bandgap widening^22^. The fact that a bandgap widening upon relaxation is observed more generally, i.e. for a larger group of amorphous materials beyond the family of phase change materials^23^,^24^,^25^, points out a possible dilemma. If resistance drift is due to bandgap widening and the latter always accompanies structural relaxation in this class of materials, there can be only little hope for finding a driftless PCM. With this work we bring the link between increasing activation energy for conduction and bandgap widening during relaxation into question. By carefully measuring the evolution of optical properties during relaxation for various phase change materials and a subsequent comparison between bandgap and activation energy for conduction, we check the degree of coupling between these two quantities.

In this paper, we present Fourier transform infrared spectroscopy (FTIR) data of Ag_4_In_3_Sb_67_Te_26_ (AIST), Ge_2_Sb_2_Te_5_ (GST) and GeTe thin films in the as-deposited amorphous state. FTIR provides an energy range, which includes the optical bandgap of all three studied materials. Therefore this method allows us to reliably investigate the evolution of the materials’ relevant optical properties during thermally accelerated relaxation at 353 K for 27 hours. The evolution of the spectra with time provides immediate insight into drift, which is in essence a phenomenon of time. This is advantageous compared with studies that are limited to the observation of the effect of annealing at various temperatures for a fixed duration^19^,^22^,^26^. Motivated by the fact that not only the bandgap, but also its temperature dependence is important when determining the activation energy of conduction (e.g. ref. 27), we present results from temperature sweeps from 353 K to 10 K before and after annealing. In the first part of this article the infrared spectra of the annealing and temperature sweep series are shown. For a quantitative analysis of the materials’ optical properties we introduce a reflectance model of the sample, which is fitted to the spectra yielding the dielectric function 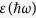 of the investigated phase change material. In addition to the main use of 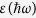, which is the determination of the optical bandgap for different temperatures and various states of relaxation, we evaluate the optical dielectric constant 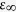 as the low-energy limit of our spectral range, i.e. 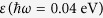. Investigating its evolution over time and temperature is a helpful input for modelling the subthreshold current-voltage characteristics of PCM cells. Since the first descriptions of conductivity in semiconductors dominated by field-enhanced emission from coulombic defects^28^, much effort was put into understanding the electrical transport in amorphous chalcogenides^29^,^30^,^31^. Because of the coulombic nature of the defect centres, the permittivity plays an important role in this kind of conductivity models. Knowledge of its dependence on temperature and relaxation therefore fixes this parameter while studying, for example, the variation of conductivity with electric field. Such experimentally determined input parameters are also crucial for simulations of advanced devices aiming for improved switching dynamics and reliability^32^. In the last part of this work we calculate the evolution of the activation energy for conduction during annealing, solely based on what would be expected from the evolution of the bandgap. The following comparison between these bandgap-based data and literature values for the activation energy answers the question how strong these two quantities are coupled during relaxation.

## Results

### Infrared spectra and dielectric function

**Figure 1:**
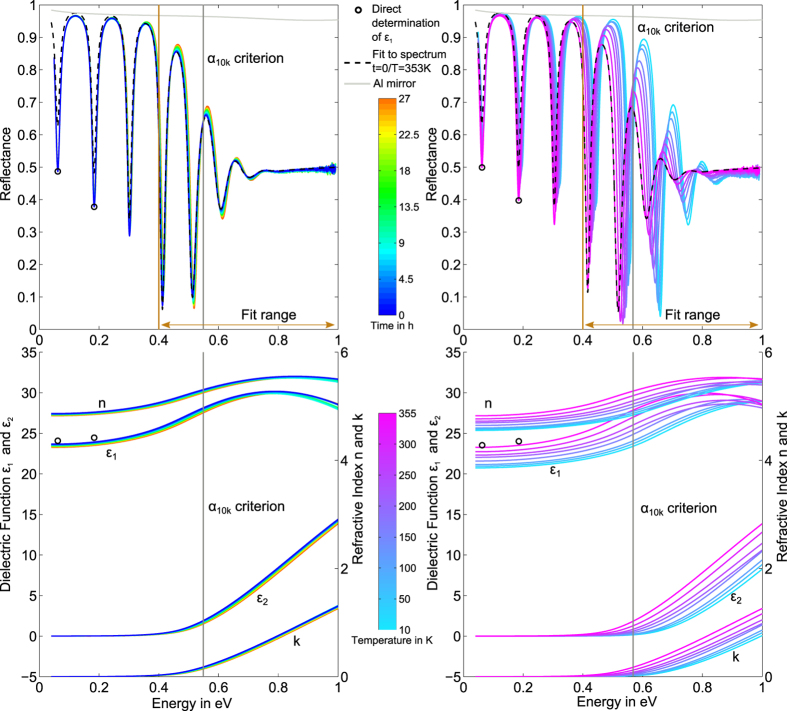
Top: Change in infrared reflectance spectra upon annealing for 27 hours at 353 K (left) and due to cooling from 353 K to 10 K (right) for amorphous Ag_4_In_3_Sb_67_Te_26_, exemplary for all three materials. Circles mark manually determined reflectance minima. The Al-reflectance forms an upper bound for the overall sample reflectance (light grey line). Each spectrum is fitted with the reflectance model including the dielectric function for the phase change material, as described in the methods. For clarity only the fit to the first spectrum during annealing at 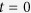 and for cooling at 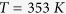 is shown (dashed black line). All fits are limited to the region of interband transitions (in this case 0.4–1 eV) Bottom: Change in the dielectric function 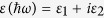 and the refractive index 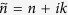, which results from fitting the spectra in the top part. Despite the limited fit region, the model still gives a good description of 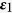 in the low energy range, as the two manually determined values for 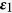 (black circles) confirm. The low energy limit of 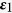 is used for a detailed investigation of 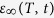 in Fig. 2. Applying the heuristic 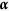
10k criterion, bandgaps from different annealing times and temperatures can be compared easily among Ag_4_In_3_Sb_67_Te_26_, Ge_2_Sb_2_Te_5_ and GeTe. Here again the bandgap is only marked for the first spectrum of each series.

The results of measuring a 1 μm thick layer of phase change material on top of an aluminum mirror with FTIR spectroscopy is shown in the top part of Fig. 1 (see also supplementary Figs 1, 2). Before analyzing the experimental data quantitatively by fitting the spectra with a model, we give a qualitative overview of their core features. All recorded spectra, regardless of annealing or temperature variation, exhibit the same general pattern, i.e. characteristic minima in reflectance up to a certain energy. These reflectance minima can be explained by multiple reflections at the sample interfaces leading to interference. For energies far below the bandgap, absorption by the phase change material is negligible and the maximum sample reflectance is only limited by the aluminum mirror. Under this condition, a simple expression can be derived to determine values of 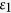 at photon energies of minimum reflectance. These values for 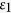 serve as reference points for later analysis. We obtain 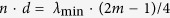 for the 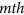 reflectance minimum at wavelength 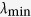, with 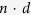 being the optical path length through the phase change layer. The imaginary part of the dielectric function 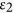 in the index of refraction 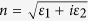 is negligible, allowing us to determine 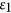 at the photon energies of minimum reflectance only based on the position of the minima 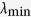 and the thickness of the film 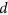. We determine values of 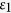 for the first two minima, as can be seen in Fig. 1, since these energies are sufficiently far away from the bandgap. For energies near and above the bandgap, interband absorption in the phase change material increases and cannot be neglected anymore. Accordingly, the height of the reflectance maxima decreases with increasing energy up to the point where interference within the phase change layer and the related oscillations in the spectra vanish completely.

Focusing on the energy range around the bandgap, the change in reflectance upon annealing and cooling is clearly visible. The points of maximum reflectance move to higher reflectance and higher energies (see e.g. the 4^th^ maximum), leading to more pronounced oscillations in the region of interband absorption. This indicates a widening of the bandgap both upon annealing and for cooling. The widening due to cooling from 353 K to 10 K is much stronger compared to the effect of annealing at 353 K for 27 hours. These qualitative observations are made likewise for GST, GeTe and AIST and might thus help in orientation during the following, quantitative analysis of the evolution of the dielectric function during annealing and cooling.

To learn more about the change in optical properties of the studied phase change materials, which are given by its dielectric function 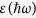, a model of the sample’s reflectance is fitted individually to each measured spectrum. Essentially, such a model contains the dielectric function and the thickness of every layer in the sample. By fitting it to a measured spectrum, we obtain the corresponding dielectric function of the phase change material. In our work, we focus on the optical absorption due to interband transitions. Here 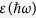 is governed by the density of electronic states (DoS) in the region around the bandgap. In our reflectance model the DoS of the phase change material is parameterised according to the OJL model^33^,^34^ (see Methods). This empirical model uses an exponential functional dependence to describe the well-known existence of localized states reaching into the bandgap of an amorphous semiconductor^18^,^35^. The energy range of the measured spectra used for fitting is limited to the region of interband transitions (0.4–1 eV for AIST, 0.5–1 eV for GST and GeTe), because those are the excitations considered by the OJL model. In this energy region, we are able to describe our data very well as can be seen for AIST in the top part of Fig. 1. This way, the dielectric function 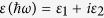 or refractive index 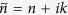 is derived for each recorded spectrum (see Fig. 1 bottom). A comparison with the earlier determined reference points for 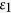 shows that the fit produces reasonable 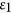 values even at photon energies below the fitted data range.

**Figure 2:**
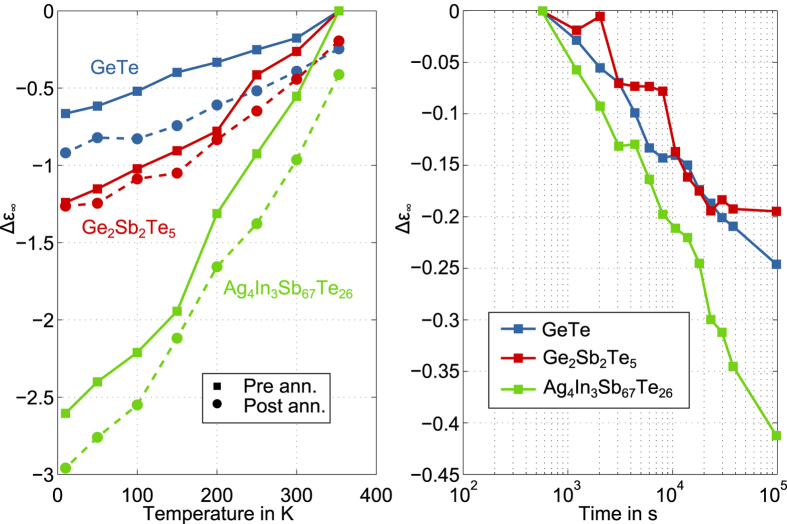
Left: Temperature dependence of 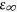 before (squares) and after annealing (dots). Right: Decrease of 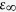 during the intermediate annealing at 353 K for 27 hours. Materials are colour-coded as indicated in the legend. The starting values of 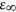 at 353 K are 23.7 (Ag_4_In_3_Sb_67_Te_26_), 17.0 (Ge_2_Sb_2_Te_5_) and 11.9 (GeTe) respectively.

After transferring the infrared spectra to the dielectric function of the studied phase change materials, we are now able to investigate the evolution of the optical properties upon annealing and cooling in detail. We start with extracting the optical dielectric constant 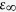 from the low energy limit of 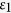 for all three materials for annealing and cooling (see Fig. 2). Looking at the temporal evolution during annealing, the observed total decrease ranges from 1.2% (GST) to 1.8% (AIST) and 2.1% (GeTe). The measured decrease is small, but still not concealed by noise and it can be described by a 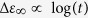 law. The temperature dependence of 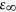 is obtained before and after annealing, the latter causing a shift towards lower values for all three materials. Cooling from 353 K to 10 K decreases 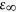 by 6.8% (GST), 10.7% (AIST) and 5.6% (GeTe).

**Figure 3:**
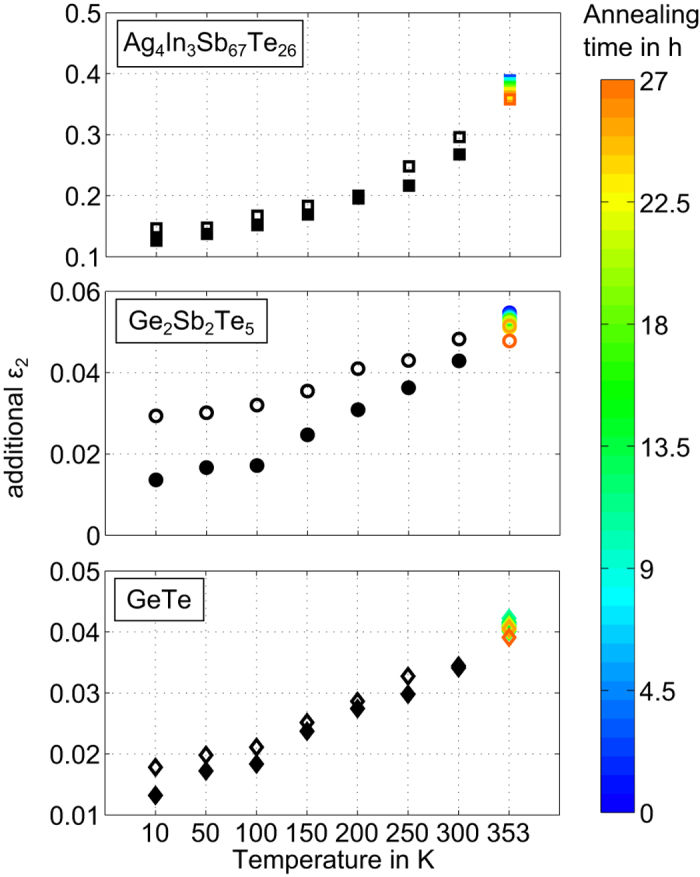
Imaginary part of the dielectric function 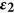 needed in addition to the OJL model in order to match the depth of the first measured reflectance minimum. This quantity’s dependence on temperature before (open black symbols) and after annealing (filled black symbols) is shown together with its evolution during the intermediate annealing at 353 K (coloured symbols).

Before turning towards the analysis of 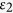, we take a look at the fit quality on the basis of Fig. 1. While the data within the region of interband transitions starting at 0.4 eV are described very well by the model, for lower energies the modelled reflectance is too high. This is especially visible at the first two minima at the example of AIST, but can be observed for GeTe and GST as well. We conclude from this overestimated reflectance that there is a contribution of absorption present in the phase change material, which is not considered in the OJL model. To find out about the evolution of this discrepancy between data and model during annealing and cooling, we determine the amount of missing absorption in the OJL model. This is done for the first measured reflectance minimum, where the discrepancy is most obvious and the influence of the interband transitions is smallest. At this point we can increase 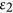 manually until the modelled reflectance is low enough to match the measured reflectance. This gives an estimation of how much additional 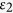 contribution would be required to describe the experimental data (Fig. 3). It reveals that quite small deviations in 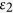 can cause pronounced differences at the reflectance minima of the measured infrared spectra. Viewing our samples as resonators for infrared light, the remarkable sensitivity of this method is a consequence of a high quality factor also manifested in the sharpness of the minima. The pronounced changes in refractive index at the phase change material’s interfaces, both with air and with the metal, effectively extend the optical path through the thin film and thus enhance the interaction of the light with the investigated material. The absolute amount of additional 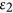 of AIST is roughly one order of magnitude larger than for GeTe and GST. The relative temperature dependences of additional 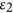 are similarly strong for all three materials with decreases ranging between 45% and 71%. Aside from this temperature dependence we observe a reduction of additional 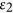 upon annealing by 5 to 8%.

### Bandgap widening during annealing and cooling

After a closer look at the low energy range in the previous section we now examine the evolution of the optical bandgap during annealing and cooling. Information about the energy dependent absorption of the phase change material is given by 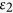 or the extinction coefficient 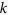 respectively. We use the widely-used, heuristic 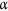
10k criterion for defining the optical bandgap^36^,^37^,^38^. Here, the bandgap energy 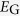 is set where the absorption 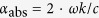 exceeds the value of 10^4^ cm^−1^. This provides a straightforward way to compare bandgaps of different relaxation states for various materials and temperatures. As already described earlier, the spectra show clear changes upon annealing and cooling indicating a bandgap widening. These changes are reflected by concomitant changes of the dielectric function and the 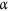
10k threshold.

**Figure 4:**
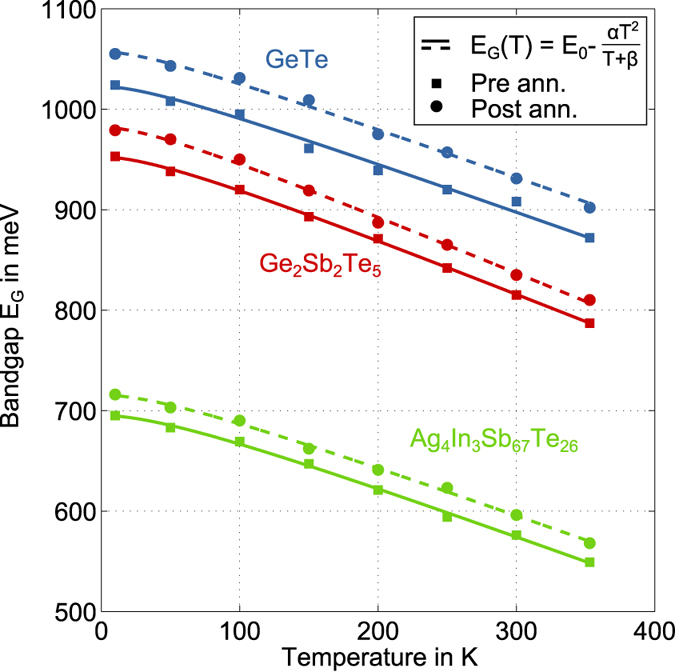
Temperature dependence of the optical bandgap of Ag_4_In_3_Sb_67_Te_26_, Ge_2_Sb_2_Te_5_ and GeTe before (squares) and after (dots) annealing at 353 K for 27 hours. Fits according to equation (1) by Varshni (see figure legend) show a transition from quadratic behaviour at low temperatures to linear behaviour at high temperatures. The resulting parameters for all fits are listed in Table 1.

We start by looking at the temperature dependent results for the optical bandgap of all three materials in Fig. 4. The bandgap widens consistently upon cooling with an increase of 26.7% for AIST, 20.8% for GST and 17.4% for GeTe, which is only slightly affected by annealing. Apart from that, we clearly observe a vertical shift between the pre and post annealing curves. A question we would like to answer quantitatively with these results is how the temperature dependence of the bandgap is influenced by relaxation. For that reason we fit the well-known Varshni model to the values of the bandgap 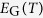
39 determined before and after the annealing.


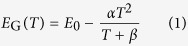



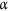
, 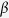 and 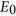 are fit parameters with 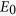 representing the bandgap at 0 K. In Fig. 4 all fits show a transition from a regime with α 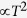 behavior into α 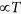 regime. Such a temperature dependence of the bandgap has been ascribed to a temperature-dependent dilatation of the lattice^40^,^41^ and a temperature-dependent electron lattice interaction^42^,^43^,^44^,^45^. Resulting fit parameters are listed in Table 1. For all three materials 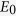 is increased after annealing, which fits to the aforementioned observation of shifts in the spectra indicating a bandgap widening during annealing. However, for 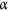 and 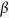 we cannot make such a clear statement, because the changes upon annealing are small compared to the confidence intervals of the fits. For GeTe the scattering of the bandgap values was even too strong to sensibly use separate 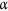 and 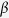 fit parameters before and after annealing. The scattering might be due to the relatively large bandgap of GeTe. Especially at low temperatures a significant portion of the energy regime characteristic for interband transitions extends beyond the experimental limits of our infrared spectra (maximum at 1 eV). Accordingly, the bandgap determination is less precise leading to higher scattering of the resulting 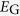 values in this case. Consequently, we fitted both 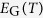 data sets for GeTe simultaneously with a common parameter for 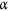 and 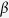 leaving only 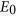 free to change upon annealing.

**Figure 5:**
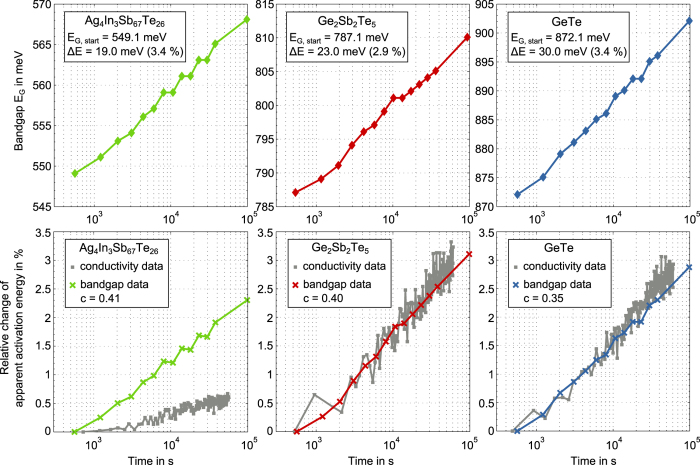
Top: Widening of the optical bandgap according to the α 10k criterion for Ag_4_In_3_Sb_67_Te_26_, Ge_2_Sb_2_Te_5_ and GeTe during annealing at 353 K for 27 hours. For all three materials the bandgap increases during annealing in a 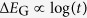 manner. Starting values, absolute and relative total changes are given in the figure legends. Bottom: The relative change of the apparent activation energy for conduction 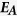 upon annealing at 353 K for Ag_4_In_3_Sb_67_Te_26_, Ge_2_Sb_2_Te_5_ and GeTe. Direct experimental data from resistance drift measurements (grey data points from^47^) are compared with what could be expected purely based on bandgap widening determined with infrared spectroscopy in this work (coloured crosses). The ratio 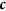 between the activation energy 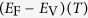 and the bandgap 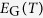 is determined at the beginning of annealing and kept fixed afterwards.

Next we apply the 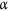
10k criterion to the data measured during annealing. As expected from the earlier qualitative examination of the spectra, the temporal evolution of 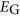 shows a monotonous increase for all three materials (Fig. 5, top). The bandgap widens in a 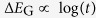 manner with total changes ranging from 19 meV (3.4%) for AIST over to 23 meV (2.9%) for GST and 30 meV (3.4%) for GeTe. To relate these changes upon annealing to the activation energy for electrical conduction, we develop an expression for the activation energy with our results for the bandgap as input. In Boltzmann approximation the activation energy of a p-type semiconductor is given by the distance between valence band edge and Fermi level 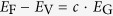 with 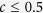
46. Motivated by the work of Oosthoek *et al.*^27^ we assume that the Fermi level maintains its relative position between valence and conduction band edge over the whole temperature range:


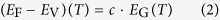


with constant c.

In practice the activation energy for conduction in amorphous semiconductors is commonly determined as the slope in a 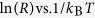 plot of the sample resistance 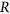 at different temperatures 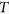. This method is based on the assumption of an Arrhenius-like temperature dependence of the resistance, which can be parameterized as


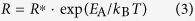


with a temperature independent prefactor 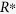 and the Boltzmann constant 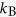. 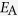 denotes the slope of the Arrhenius plot


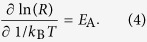


However, when a temperature dependence of the activation energy must be considered, 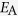 is not necessarily equal to 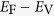. In what follows we thus refer to 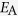 as the ‘apparent activation energy’ and hereby distinguish it from the earlier introduced activation energy 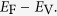 To develop an expression for 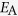 based on the temperature dependent activation energy, we recalculate the slope of the Arrhenius plot with 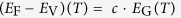
instead of 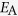.


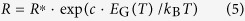


leads to


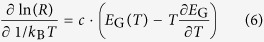


Equating the right hand sides of equations 4 and 6 shows that the correction term 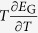 is necessary when expressing the apparent activation energy 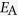 in terms of the bandgap 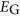 (equation 7).


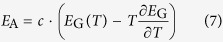


Wimmer *et al.* performed van-der-Pauw measurements on as-deposited thin films of AIST, GeTe and GST and investigated the change of the apparent activation energy for conduction 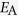 during annealing at 353 K for 16 hours^47^. To compare their results with what we would expect based on our bandgap data for the exact same materials and annealing temperature, we need to subtract 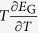 from the bandgap values at 353 K. 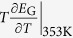 is given in Table 1 together with the outcome of the fitting of the Varshni model to the temperature dependence of the optical bandgap 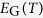 described earlier. In the case of AIST and GST, where we observe a change in 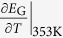 during annealing, we interpolate this change linearly over the time span of annealing. The constant 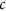 in equation 7, which is defined as the fixed ratio between activation energy and bandgap (equation 2), is determined at the beginning of the annealing period. The relative change of the apparent activation energy 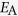 during annealing, as it results from the conductivity measurements of Wimmer *et al.*, is compared with the relative change of 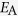 deduced from our bandgap data in the bottom part of Fig. 5. In the case of GST and GeTe the temporal change in 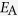 can be fully ascribed to the widening of the bandgap reaching roughly 3% after 27 hours. For AIST a slightly lower increase (2,3%) is deduced from the bandgap data. Most strikingly, however, the actual drift of the apparent activation energy for conduction measured electrically is more than 4 times smaller compared to what would result from a mere widening of the bandgap.

## Discussion

Describing the DoS of our amorphous semiconductors with parabolic band edges and exponential bandtails allows an excellent fitting of the interband transitions in the measured infrared spectra. The small but significant discrepancy between data and model at lower energies might be related to defect states in the bandgap. In this case, the additional amount of 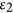 increasing with temperature would imply a growing absorption involving defect states. An explanation to this behavior could be an increasing number of occupied starting states and/or unoccupied end states for optically induced electronic transitions, caused by a dependence of the Fermi distribution on temperature. Following this line of thought the decrease of additional 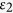 during annealing could be interpreted as shrinking absorption by defect states, caused by a non-reversible decrease in the number of defect states upon relaxation. This way, both the reversibility of temperature change and the non-reversibility of resistance drift can be qualitatively explained. A decreasing number of mid-gap states during relaxation was already reported based on density functional theory calculations for GeTe by Raty *et al.*^22^. However, neither their experiments with photo thermal deflection spectroscopy (PDS) nor other PDS measurements on phase change materials published so far have provided any indication of states in the bandgap^19^,^22^,^38^. Such defect states have been necessary to describe the electrical properties of GeTe^48^. The lacking sign of those defect states in PDS was argued to be due to optical capture coefficients for these states being approximately 40 times smaller than for the bandtails and extended states. The capability of FTIR spectroscopy to provide evidence for such defect states in contrast to PDS reveals an advantage of FTIR measurements. While PDS can detect extremely small amounts of energy from light absorbed while passing through a thin film, the experimental configuration utilized in FTIR becomes highly sensitive through multiple reflections and interference. While the computer simulations by Raty *et al.* were limited to GeTe^22^, our experimental results do not only substantiate the existence of defects in the bandgap and their reduction during drift for this material, but also allow an extension of this statement to the family of Sb_2_Te-based phase change materials.

Regarding the optical dielectric constant 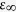, we observe a slight decrease over time during annealing for all three materials. Together with the temperature dependence of 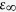 these results can serve as a valuable input for Poole-Frenkel-like conductivity models^32^. The measured decrease in 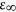 is in line with density functional theory calculations for GeTe from Raty *et al.*^22^, which fits to their conclusion that characteristic electronic properties of the amorphous state drift away from those of the crystal during relaxation. Once again, our quantitative, experimental results help to confirm their statement and even expand it to a material that does not contain any Germanium. The same applies to bandgap widening during relaxation, which was interpreted as a consequence of enhanced Peierls-like distortions, manifested in GeTe by the disappearance of tetrahedral coordination around Ge atoms and the formation of threefold coordination^22^. Our measurements confirm bandgap widening as a fingerprint of relaxation and moreover support the belief of Raty *et al.*, that the underlying mechanisms responsible for aging in amorphous GeTe could act similarly in Te-based phase change materials like the Sb_2_Te-family.

In addition to confirming existing simulations and expanding their statements to other materials by means of quantitative results, our work provides a new, remarkable finding regarding the technologically highly relevant link between structural relaxation and its electronic effects. Based on observations for AIST we show that the drift of the apparent activation energy for conduction can be largely decoupled from the widening of the bandgap. A conceivable explanation would be a shift of the relative position of the Fermi level between the band edges, partially compensating for the increase in activation energy due to the pronounced bandgap widening. Such a Fermi level shift could be caused by a beneficial decay of defects in the bandgap upon annealing. A stronger reduction in absorption due to defect states in AIST compared to GST and GeTe would be in favour of such a hypothesis. Future studies may be motivated to unravel the exact underlying mechanisms leading to the observed decoupling and even to develop ways to manipulate it, potentially by defect design. However, already based on the results presented in this article we can draw a strong conclusion: Even if a widening of the bandgap upon relaxation cannot be avoided, the identification of alternative phase change materials with much reduced drift of the electrical conduction is proven to be achievable.

## Methods

### Sample preparation

Each sample consists of a 1 μm thick layer of phase change material, which is deposited on top of a 200 nm thick aluminum layer on a glass substrate (2 cm × 2 cm). The aluminum layer acts as a mirror because of its 98% reflectance for infrared wavelengths, maximizing the sample reflectance and the detector signal. All films have been deposited by direct current sputtering with an LS 320 von Ardenne system at a background pressure of 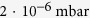 with 20 sccm Argon flow operating in constant power mode (20 W) using stoichiometric targets of 99.99% purity.

### Measurement procedure

A Bruker IFS 66v/S FTIR spectrometer is used for measuring infrared reflectance spectra together with a Konti Spektrum A cryostat by Cryovac. A silicon carbide rod acts as radiation source providing photons in an energy range of 0.025–1 eV. The actually measured quantity is the signal from the DTGS intensity detector as a function of the interferometer mirror position. The signal vs. frequency spectrum results from a Fourier transformation of that interferogram. The sample under test is placed in a 10^−5^ mbar vacuum to reduce any additional light absorption by gases in the sample chamber as well as significant condensates on the surface of the cooled sample. The cryostat is cooled with liquid helium and covers a 10 K to 400 K temperature range by the use of a heater and a temperature sensor. The error of temperature measurement was tested with an additional sensor and found to be below 1.5 K. Every analyzed spectrum results from averaging over 50 spectra to obtain the best signal to noise ratio, which leads to an overall measurement duration of 90 s. One measurement series includes two temperature sweeps separated by an annealing period. The first temperature sweep with spectra recorded at 10 K, 50 K, 100 K, 150 K, 200 K, 250 K, 300 K and 353 K is followed by 14 spectra recorded during the annealing periods at 353 K for 27 hours. The second temperature sweep identical to the first sweep is performed directly after the annealing period. To account for frequency dependencies in the optical components of the setup, every reflectance spectrum is divided by a reference spectrum, which is measured with a 50 nm thick gold layer on glass providing nearly 100% reflectance in the used spectral range. Such a reference spectrum is recorded *in-situ* immediately before each measurement to exclude artifacts due to intensity fluctuations.

### Modelling the sample reflectance

The reflectance model for the entire sample contains the dielectric function 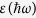 for the material of each layer and the respective layer thickness^49^. In our case the sample consists of a 1μm thick phase change material layer on top of a 200 nm thick aluminum layer, deposited on a glass substrate. The properties of the glass substrate are neglected because of the high reflectance of the aluminum layer. The dielectric function of aluminum was determined by measuring the reflectance spectrum of a bare aluminum layer on top of a glass substrate. Fitting this spectrum with a Drude model provides 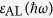
50. All fitting is done within the numerical computing environment MATLAB. The dielectric function of the phase change material 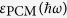 is determined by fitting the reflectance model for the layer stack to the measured spectra. Because we limit our analysis to interband transitions, the model for 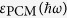 is given by the density of states (DoS) around the bandgap and the probability for transitions between these states. The used model for the DoS is motivated by the description of the DoS of amorphous semiconductors by Mott^18^, which introduces localized band tail states reaching into the bandgap. O’Leary *et al.* developed an empirical model for the optical absorption accounting for such a distribution of electronic states^33^,^34^. This so-called OJL model is comprised of a square-root functional dependence in the band region and an exponential functional dependence in the tail region. The conduction- and valence band DoS 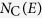 and 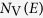 are given by equation 8 and 9.


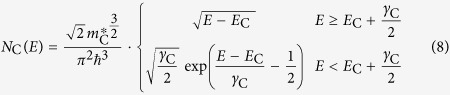



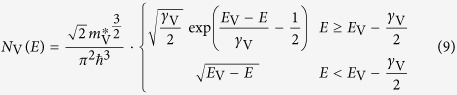



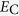
 and 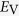 mark the conduction and valence band edge respectively. 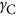 and 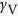 are measures for how far the bandtails extend into the bandgap. Following O’Leary *et al.*^34^ from this DoS we obtain the joint density of states 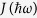, which is related to the absorption coefficient 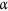 by 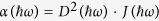 with 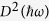 being the optical transition matrix element. Because the exact functional dependence of 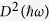 remains unknown, we make the usual assumption that 
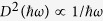
34,51,52,53,54. Using 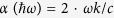, we calculate the extinction coefficient 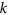 in the refractive index 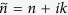, in which 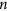 is related to 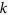 by the Kramers-Kronig relation. Finally, 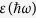 is derived from 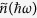, with the optical dielectric constant 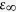 as the low energy limit of 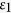.

## Additional Information

**How to cite this article**: Rütten, M. *et al.* Relation between bandgap and resistance drift in amorphous phase change materials. *Sci. Rep.* **5**, 17362; doi: 10.1038/srep17362 (2015).

## Supplementary Material

Supplementary Information

## Figures and Tables

**Table 1:** Fitting the Varshni equation 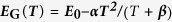 to the bandgap according to the α 10k criterion (Fig. 4) yields one set of parameters before and one after annealing for Ag_4_In_3_Sb_67_Te_26_ and Ge_2_Sb_2_Te_5_.

	Ag_4_In_3_Sb_67_Te_26_		Ge_2_Sb_2_Te_5_		GeTe	
	pre ann.	post ann.	pre ann.	post ann.	pre ann.	post ann.
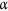 in meV/K	0.508 ± 0.013	0.499 ± 0.086	0.555 ± 0.009	0.575 ± 0.017	0.492 ± 0.027	
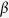 in K	77.37 ± 9.03	73.28 ± 10.41	64.95 ± 4.86	56.39 ± 9.03	53.68 ± 18.74	
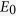 in meV	695.3 ± 2.2	715.6 ± 2.6	952.6 ± 1.5	982.1 ± 3.0	1022.8 ± 6.7	1057.3 ± 6.7
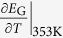 in meV/K	−0.492 ± 0.009	−0.484 ± 0.011	−0.542 ± 0.007	−0.564 ± 0.014	−0.483 ± 0.021	

In view of the larger scattering of the 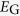 values for GeTe, we only allowed 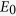 to change upon annealing. The fit parameters 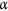 and 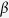 had each to describe the pre and post annealing data sets of GeTe with one constant value. The derivative of 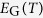 at 353 K is calculated for each fit in order to compare our findings with electrical measurements of the apparent activation energy 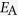 according to equation (7).
