# Supplementary material for: Relation between bandgap and resistance drift in amorphous phase change materials
Source: Sci Rep. 2015 Dec 1;5:17362. doi: 10.1038/srep17362 (PMC4664898; doi:10.1038/srep17362)
Supplement: Supplementary Information [file srep17362-s1.pdf]

Relation between bandgap and resistance drift in amorphous phase change materials

Martin Rütten[1,2], Matthias Kaes[1], Andreas Albert[1], Matthias Wuttig[1] and Martin Salinga[1]

[1] Institute of Physics 1A, RWTH Aachen University, Sommerfeldstrasse 14, 52074 Aachen, Germany

[2] IBM Research - Zurich, Säumerstrasse 4, 8803 Rüschlikon, Switzerland

Supplementary Information

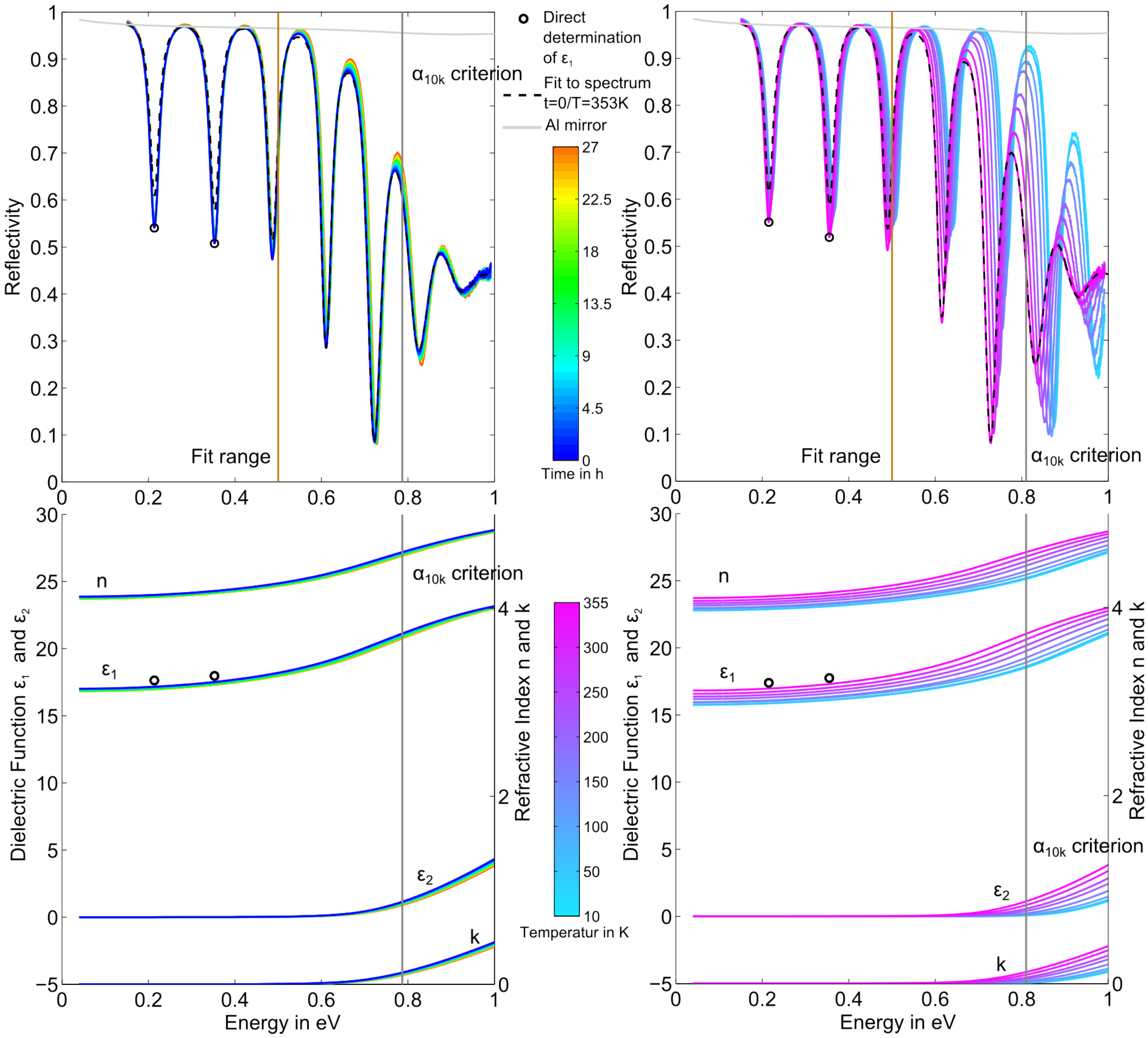

Supplementary Figure 1: Change upon annealing and cooling in infrared reflectivity spectra and the resulting dielectric function for amorphous $Ge_2Sb_2Te_5$ in analogy to figure 1 of the main text.

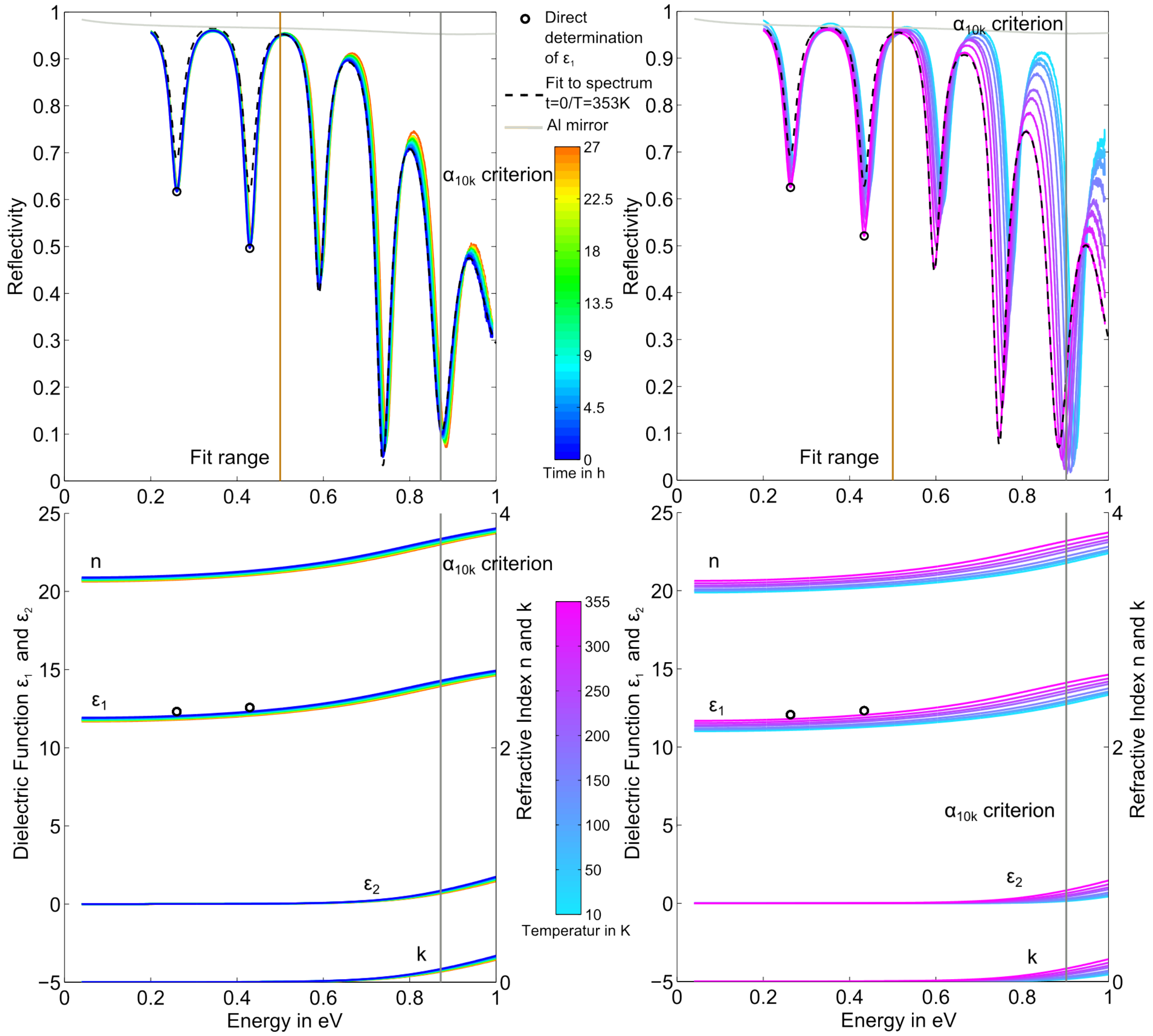

Supplementary Figure 2: Change upon annealing and cooling in infrared reflectivity spectra and the resulting dielectric function for amorphous GeTe in analogy to figure 1 of the main text.
